# On the Application of Respirometric System on Hazelnut Samples to Predict Aflatoxin Production

**DOI:** 10.3390/toxins18090385

**Published:** 2026-09-06

**Authors:** Francesca Bosco, Chiara Mollea, Gabriele Baldissone, Micaela Demichela, Davide Fissore

**Affiliations:** Department of Applied Science and Technology, DISAT, Politecnico di Torino, C.so Duca Degli Abruzzi 24, 10129 Torino, Italy; chiara.mollea@polito.it (C.M.); gabriele.baldissone@polito.it (G.B.); micaela.demichela@polito.it (M.D.); davide.fissore@polito.it (D.F.)

**Keywords:** aflatoxin, hazelnuts, respirometric sensor, respiratory quotient, autoregressive model

## Abstract

In the present work, the correlation between the respirometric activity and the aflatoxin synthesis of the hazelnut microbiota has been investigated. Microcosms containing a monolayer of hazelnuts were maintained at controlled conditions in terms of temperature and humidity. To monitor microbial growth, CO_2_ was evaluated along with the specific O_2_ uptake rate, RO_2_, measured by a respirometric sensor, usually applied in the evaluation of soil microbial activity. The respiratory quotient (RQ) was calculated and a linear relationship between RQ and produced aflatoxins was obtained. Depending on incubation time, four main clusters of RQ values were identified: for higher incubation time (15–40 days) RQ was lower than 2 and an aflatoxin amount of 0.31–0.35 μg/kg was observed. CO_2_, O_2_, and aflatoxin data have been used to develop an autoregressive model and excellent agreement between measurements and estimations was obtained. In most cases, an error lower than 1% on the estimated value of the mycotoxin amount revealed the accuracy of the evaluation of the RQ. The present work represents the first attempt to develop an effective method, fast and low-cost, for the in-line monitoring of mycotoxin contamination.

## 1. Introduction

Hazelnut production represents one of the most important segments of the global nut industry. According to the most recent data available in the Food and Agriculture Organization Statistics (FAOSTAT) database, the main countries involved in the hazelnut supply chain are Turkey, with 765,000 tons, covering about 64% of global production, and Italy, with 98,670 tons, contributing nearly 8% of global demand [1]. The growing interest in hazelnuts is probably due to their nutritional properties (i.e., nutritional value, fatty acid profile, and tocopherols content), which allow hazelnuts to fit the definition of functional food [2]. The high consumption of hazelnuts, as a major ingredient in bakery and confectionery products, has consolidated their economic importance worldwide. However, the increasing consumption has also raised concerns regarding consumer health because of the potential occurrence of mycotoxin contamination [3].

Mycotoxins represent the main risk associated with the hazelnut supply chain; they are produced by the secondary metabolism of filamentous fungi, primarily belonging to the genera *Aspergillus*, *Penicillium* and *Fusarium* [4].

The presence of filamentous fungi on hazelnuts does not necessarily indicate the presence of mycotoxins, since the optimal conditions for fungal growth do not always coincide with those favorable for mycotoxin biosynthesis [5]. Nevertheless, fungal colonization should be considered a warning. Furthermore, the ability of mycotoxigenic fungi to adapt to climate change may alter their geographical distribution and affect the pattern of mycotoxin contamination [6].

Under suitable temperature and humidity conditions, fungal spores present on the hazelnut surface can germinate and synthetize mycotoxins throughout all stages of the food production chain. Aflatoxins (AF) are the most relevant mycotoxins in hazelnuts. Aflatoxigenic fungi can grow over a wide range of temperature (5–35 °C) and humidity (70–90%) values, making mycotoxin contamination a widespread concern [7]. The minimum and maximum temperature and water activity values reported for the main foodborne mycotoxigenic species have been reviewed by Sautour et al. [5]. To ensure food safety during storage conditions, the moisture content should be about 10% (wet basis) for inshell hazelnuts and 6% for kernels [8,9].

Once formed on foods, mycotoxins are very difficult to eliminate because of their relative stability under thermal treatments and therefore able to persist following conventional cooking conditions or food industry processes such as pasteurization [3].

Even if mycotoxin contamination is ubiquitous, its harmful effects are particularly severe in developing countries, where adequate monitoring, detection, and decontamination methods are often lacking. To protect human and animal health, regulatory authorities in many countries have established maximum limits for specific mycotoxins in food, feed, and commodities. Nevertheless, specific population subgroups may be susceptible because of their higher consumption of contaminated products [3,10].

Considering that fungal growth and mycotoxin contamination can occur at every stage of the food supply chain and they may accumulate over time, the evaluation of mycotoxin, not only on hazelnuts, becomes mandatory [7].

In 2023, Salvatore et al. [11] reported a data collection on mycotoxins detected in hazelnuts, together with the corresponding mycotoxigenic fungal species. This work highlighted the large variety of mycotoxins without focusing on a single or specific groups such as aflatoxins, as reported in previous studies. The possible co-occurrence of multiple mycotoxins in hazelnuts forces the development of multi-mycotoxin analytical methods to monitor a broad range of compounds. The complex composition of the hazelnuts (i.e., high fat content) combined with the relatively low mycotoxin concentrations makes the analysis a challenging task. In fact, multiple analytical methods must be employed on the same sample to achieve reliable data. For these reasons, the mycotoxin determination in food is very difficult and time consuming.

In this work, the authors underlined the necessity to develop suitable methods for the simultaneous detection of a wide range of mycotoxins. Several technological approaches, including irradiation, ultrasound, plasma treatments, and chemical decontamination, have been explored to reduce AF contamination in hazelnuts. However, these technologies often suffer from limitations related to cost and industrial scalability. Consequently, preventive measures and continuous monitoring remain the most effective and economically sustainable solutions [12].

In a previous work, Mylona and Magan demonstrated that *in situ* measurements of CO_2_ production may be useful for predicting toxin synthesis and accumulation in oat storage [13]. Subsequently, Garcia-Cela et al. [14] evidenced a correlation between CO_2_ production and mycotoxin accumulation in stored shelled peanuts or those either naturally contaminated or artificially inoculated with *A. flavus* conidia. Moreover, the relative risk of Aflatoxin B1, AFB1, contamination during 7 days of peanut storage by monitoring CO_2_ production was discussed. Based on these findings, Mejia et al. [15] suggested that CO_2_ production could be adopted as an early indicator of fungal colonization and mycotoxin production, supporting dynamic risk assessment throughout the supply chain of hazelnuts, through continuous monitoring in different nodes.

Among the different chemical physical factors influencing fungal growth and mycotoxin production on food, it is necessary to distinguish between pre-harvest and post-harvest conditions. During the pre-harvest phase, as reported by [16], temperature, relative humidity, rainfall, and kernel water activity (a_w_) have been identified as the main environmental factors affecting fungal growth. During storage, temperature and a_w_ are considered the two major limiting factors on *A. flavus* growth and aflatoxins production [17].

The aim of the present work was to demonstrate the correlation between the respirometric activity of indigenous filamentous fungi on hazelnuts (healthy hazelnuts and brown marmorated stink bug-BMSB damaged ones) and the aflatoxin synthesis. The CO_2_ production was used as an early indicator of fungal contamination. To this end, a closed system (i.e., microcosms with two different geometry), containing a monolayer of hazelnuts, was used under controlled temperature and humidity conditions to promote biomass development and mycotoxin synthesis. A respirometric sensor system, usually applied for the assessment of soil microbial activity, was used to monitor fungal growth on hazelnuts and correlate respirometric activity with mycotoxin production.

The present work has to be considered as the first attempt to develop a rapid, reliable, and cost-effective method for the in-line monitoring of mycotoxin contamination in hazelnut.

A simple relationship between the respirometry quotient (RQ), defined as the ratio between CO_2_ produced and O_2_ consumed, and the amount of aflatoxin produced was established from the measured respiratory data. Moreover, the possibility of estimating the evolution of mycotoxin production was investigated. An autoregressive model [18,19] was used for this purpose; the parameters of this model are identified from experimental values of CO_2_ and O_2_ in a given time interval and then, the model was used in different cases, for both one-step-ahead prediction and the long-term prediction, showing good accuracy.

## 2. Results and Discussion

### 2.1. Hazelnut Characterization

Hazelnut characterization was carried out for all tested types: BMSB damaged, whole or broken (D-W and D-B), and healthy, either in shell or as kernels only (H-S or H). Table 1 summarizes the average values obtained for the evaluated parameters. As expected, whole kernels (D-W and H) showed the highest weight and dimensions, with a Dp between 11.6 and 12.1 mm and a Ψ close to 1.0 (Ψ equal to 1.0 indicates spherical nuts) [2]. For broken hazelnuts (D-B), the weight was approximately 50% lower than that of whole kernels. Dimensions A and B were about 1 mm smaller than those of whole kernels, while thickness (C) was reduced by half; this was also reflected in the Dp, which was equal to 8.9 mm, and in Ψ, which was 26% lower than 1.0. For this type of hazelnut, breakage introduced variability in size and in the assessed parameters exceeding 15%. Finally, for hazelnut with shell (H-S), the weight and the three dimensions were approximately 50% and 30% higher, respectively, than those of whole kernels without shells. The pH of kernels and in shell nuts was similar for almost all the samples, ranging from 6.0 to 6.2.

### 2.2. Microcosm Tests with Different Types of Hazelnuts

In cylindric microcosm (Jm) tests, different types of hazelnuts were used: healthy in shell (H-S), shelled healthy (H) and BMSB (*Halyomorpha halys* Stal) damaged hazelnuts, either whole (D-W), broken (D-B), or as a mixture of the two (D-WB), which have become a worldwide threat to hazelnut yield [20]. All microcosms were prepared in jars (see Figure 10 in Section 4) with a monolayer of hazelnuts and maintained at 40% R.H. and 25 °C, as described in Section 4 (see Section 4.2.1).

**Figure 1:**
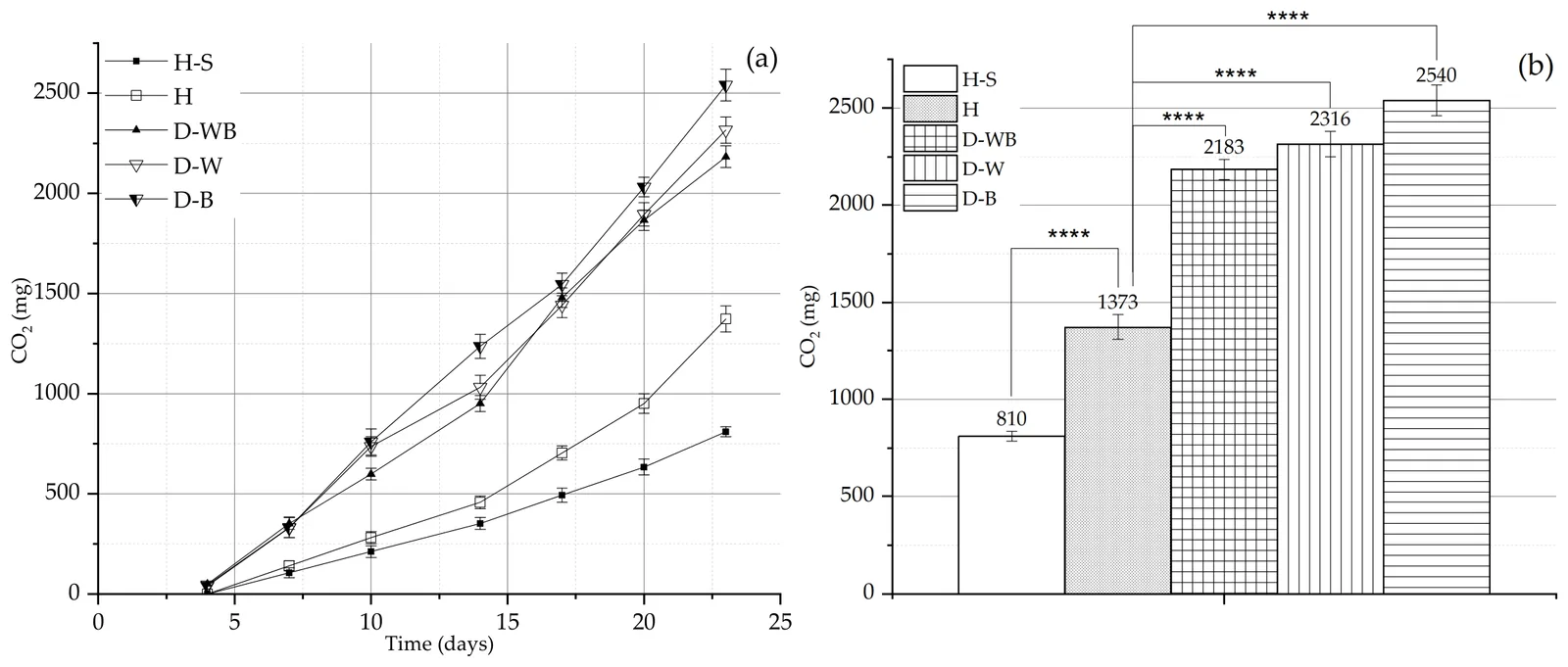
(**a**) CO_2_ production with different types of hazelnuts. (**b**) Total CO_2_ produced at the 23rd day of incubation. “****” for *p* < 0.0001.

In the first test, the growth of indigenous fungi was evaluated by measuring CO_2_ production at different incubation times. As can be observed in Figure 1a, all microcosms exhibited respirometric activity from the 7th day of incubation onward. D-B, D-W and D-WB showed comparable CO_2_ production (in the range of 330–350 mg), whereas H and H-S, exhibited significantly lower CO_2_ production during the same period (H had a cumulative CO_2_ value of 114 mg and H-S of 77 mg). At the end of the incubation period (23 days), the highest total CO_2_ production was observed for D-B (2540 mg), probably due to the higher exposed surface area with respect to D-W (2316 mg) and D-WB (2183 mg) (Figure 1b).

**Figure 2:**
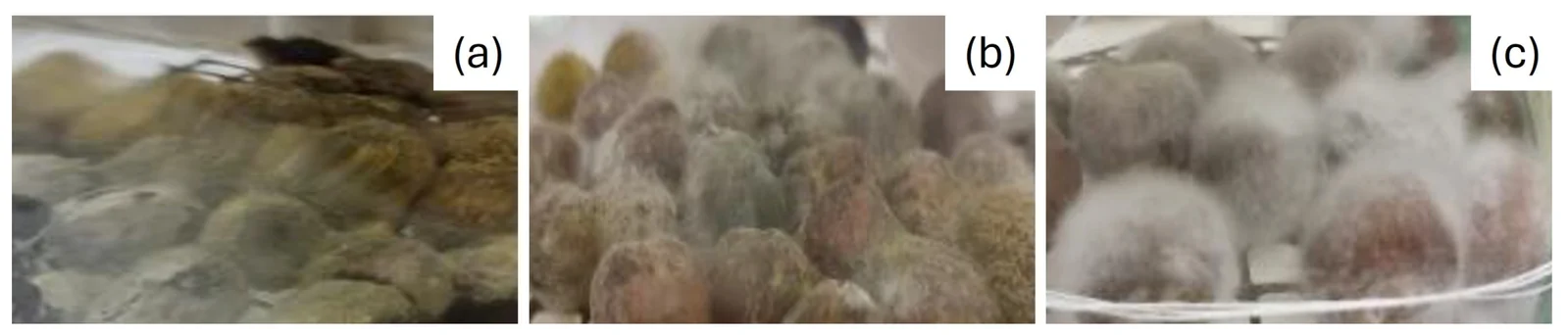
Biomass growth in microcosm at 23 days of incubation: (**a**) D-W, (**b**) H, and (**c**) H-S hazelnuts.

As regards healthy hazelnut in shell (H-S) and without shell (H), the total CO_2_ produced after 23 days of incubation (810 and 1373 mg, respectively) was lower than that obtained for BMSB damaged hazelnuts, probably due to a lower microbial contamination. The respirometric activity observed in the presence of H-S suggested the possible presence of ligninolytic fungi, capable of growing on the lignocellulosic shell. At the end of the incubation period, the pure mycelium observed on H-S (Figure 2c) and the mixed biomass present on shelled hazelnuts, D-W (Figure 2a) and H (Figure 2b), support the previous hypothesis.

For H-S healthy hazelnuts in shell, the specific CO_2_ production rate, calculated at the end of the incubation period (from the 20th to the 23rd day) was the lowest, at 0.067 mg CO_2_/g∙h. In contrast, similar values were calculated for all the other hazelnut types (H, D-WB, and D-B), ranging from 0.12 to 0.19 mg CO_2_/g∙h (see Table 2). On the 23rd day, H hazelnuts were characterized by the highest total aflatoxin content (0.30 µg/kg_DW_), whereas significantly lower values were observed for D-B and D-W (0.15 µg/kg_DW_ and 0.064 µg/kg_DW_, respectively).

Given the highest mycotoxin concentration observed in the microcosm containing healthy hazelnuts, the influence of aeration time on CO_2_ and mycotoxins production was subsequently evaluated using H hazelnuts.

### 2.3. Microbiota of Healthy Hazelnuts

The microbiota of H hazelnuts was evaluated before the microcosm set-up on MEA plates supplemented with or without chloramphenicol. As evidenced by MEA plates incubated for 7 days at 25 °C, both bacteria and fungi were present on the hazelnuts (Figure S1a), whereas, in the presence of chloramphenicol, as expected, only colonies of filamentous fungi were observed (Figure S1b).

During microcosm incubation, together with the respirometric analysis, the most common fungal colonies were isolated. It was observed that different strains developed within the same microcosm and that, over time, one species prevailed over another. Moreover, in some cases, the most frequent colonies were not detected before incubation.

**Figure 3:**
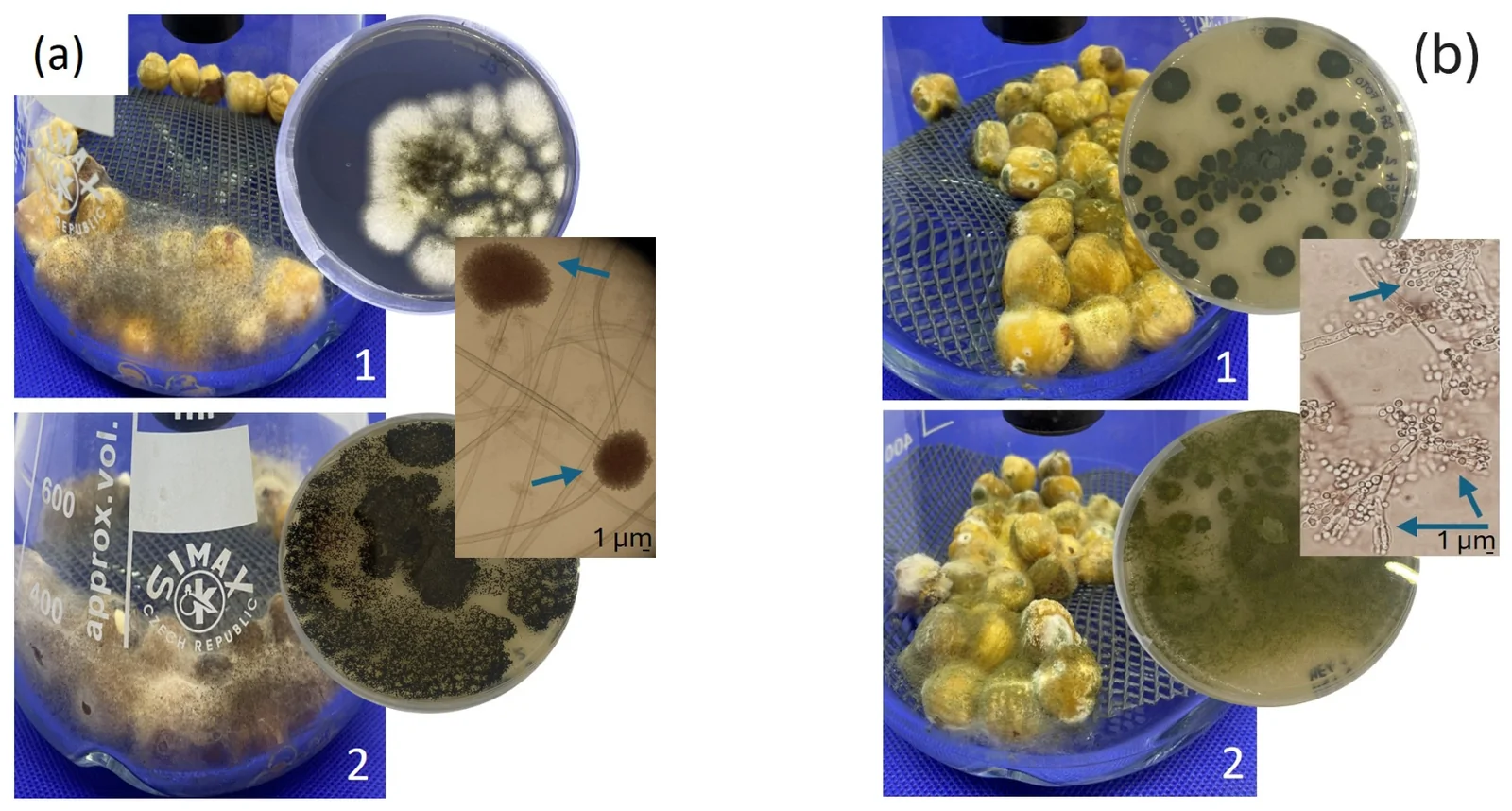
Isolated strains from different microcosms. Isolated *Aspergillus* strains at 22 and 59 days (**a**), 1 and 2, respectively. Isolated *Penicillium* strains at 9 and 25 days (**b**), 1 and 2, respectively.

The hazelnut monolayer was progressively colonized, and some strains appeared in different microcosms, although at different times. A few examples are reported in Figure 3 for D-W, H, and H-S hazelnuts; at 22 and 59 days, *Aspergillus* strains were isolated (see Figure 3(a1,a2), respectively). In Figure 3b, *Penicillium* strains were isolated after 9 and 25 days of incubation in two different microcosms (see Figure 3(b1,b2)). *Penicillium* and *Aspergillus* species are reported in the literature as mycotoxin producers [4] and can be found in all varieties of dried hazelnuts from different geographical areas [21].

### 2.4. Tests with a Different Microcosm Geometry

In the environmental field, respirometric sensor systems, such as the one used in this study, represent an essential tool for determining soil microbial activity. In the present work, an attempt was made to apply the same system to evaluate microbial contamination in food, and this entailed the use of a different microcosm geometry. To this end, as will be described in Section 4 (see Section 4.2.2 and Figure 11), comparison with the previously used microcosm was mandatory. Three different microcosm configurations (Jm—cylindric microcosm, Cm—cone microcosm, and Sm—cone microcosm with the respirometric sensor) were prepared using H hazelnuts and maintained at 25 °C and 40% R.H. in the dark.

The geometry of the microcosm and the presence of the respirometric sensor did not influence the CO_2_ production rate and, consequently, the biomass growth rate. In fact, both the trend and the amount of produced CO_2_ were comparable among the different configurations (ranging from 81 to 82.4 mg/g_DW_ CO_2_ after 11 days of incubation) (Figure S2). Statistical analysis confirms this observation, as the *p* values at each incubation time were higher than 0.05. Table 3 shows the cumulative CO_2_ values and the specific CO_2_ production rate. A decrease can be observed until the 9th day of incubation, followed by a slight recovery, probably due to the 2-day aeration interval following the previous 3-day interval. This result suggested an influence of aeration frequency on CO_2_ production, as discussed in Section 2.5.

Moreover, during incubation, biomass development was observed over time in all microcosm types in a comparable way (biomass at 11th day is shown in Table 3). Considering these results, in the following tests, the most suitable microcosm geometry was used according to the measured parameter (i.e., CO_2_ production and/or O_2_ consumption).

### 2.5. Microcosm Tests on Healthy Hazelnuts to Evaluate the Influence of Aeration Time

**Figure 4:**
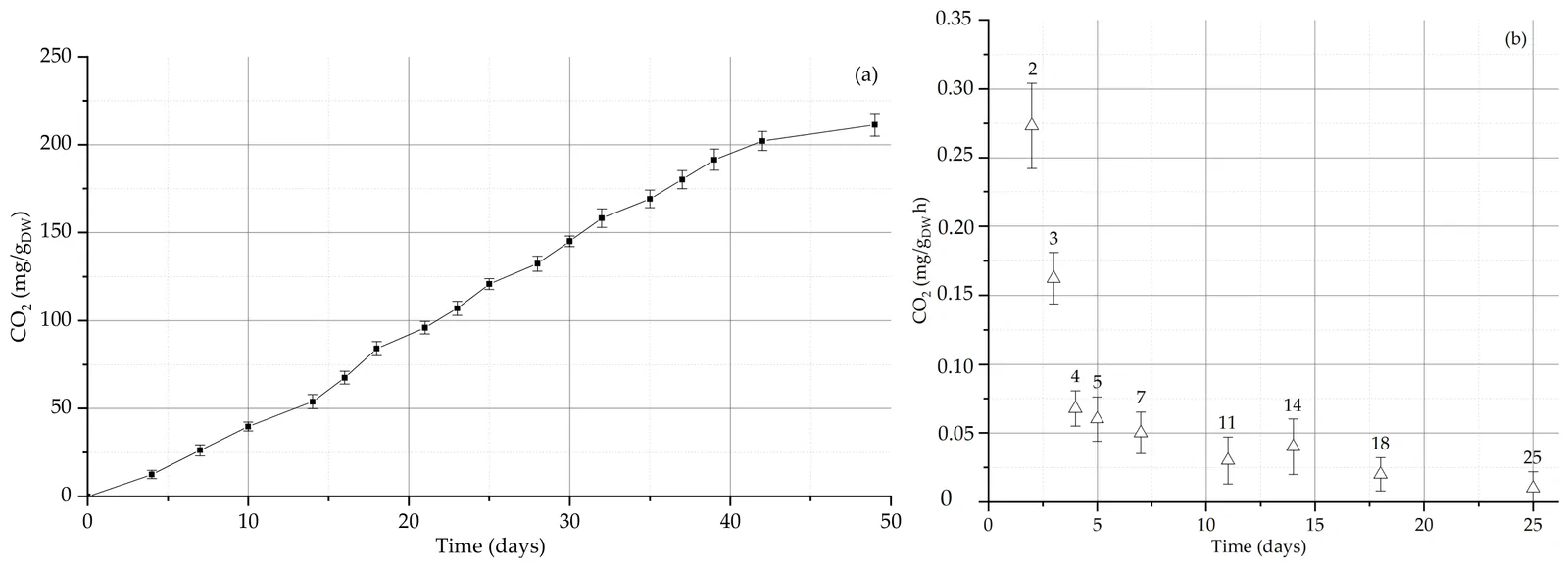
(**a**) Cumulative curve of produced CO_2_. The arrows in the graph indicate the reduction in velocity attributable to the higher aeration Δt. (**b**) Dependence of specific CO_2_ production rate from aeration frequency during the incubation with different aeration intervals (reported above the values).

Tests were carried out in cone microcosm (Cm) using H hazelnuts, at 25 °C and 40% R.H., over a period of 49 days. The obtained CO_2_ production is shown in Figure 4a. The cumulative CO_2_ value (mgCO_2_/g_DW_) increased throughout the whole test; however, the growth rate was clearly influenced by the aeration interval. In fact, Figure 4b shows the dependence of the specific CO_2_ production rate on aeration frequency. For a 2-day interval, an average value of 0.27 mg/g_DW_h was observed, which decreased to 0.16 mg/g_DW_h with a 3-day aeration interval and to 0.07 mg/g_DW_h with a 4-day interval. Beyond this frequency, the specific rate continued to decrease, although with contained variability, reaching values between 0.01 and 0.06 mg/g_DW_h, even for substantially different aeration intervals, ranging from 5 to 25 days.

### 2.6. Microcosm Tests with Respirometric Sensor System

**Figure 5:**
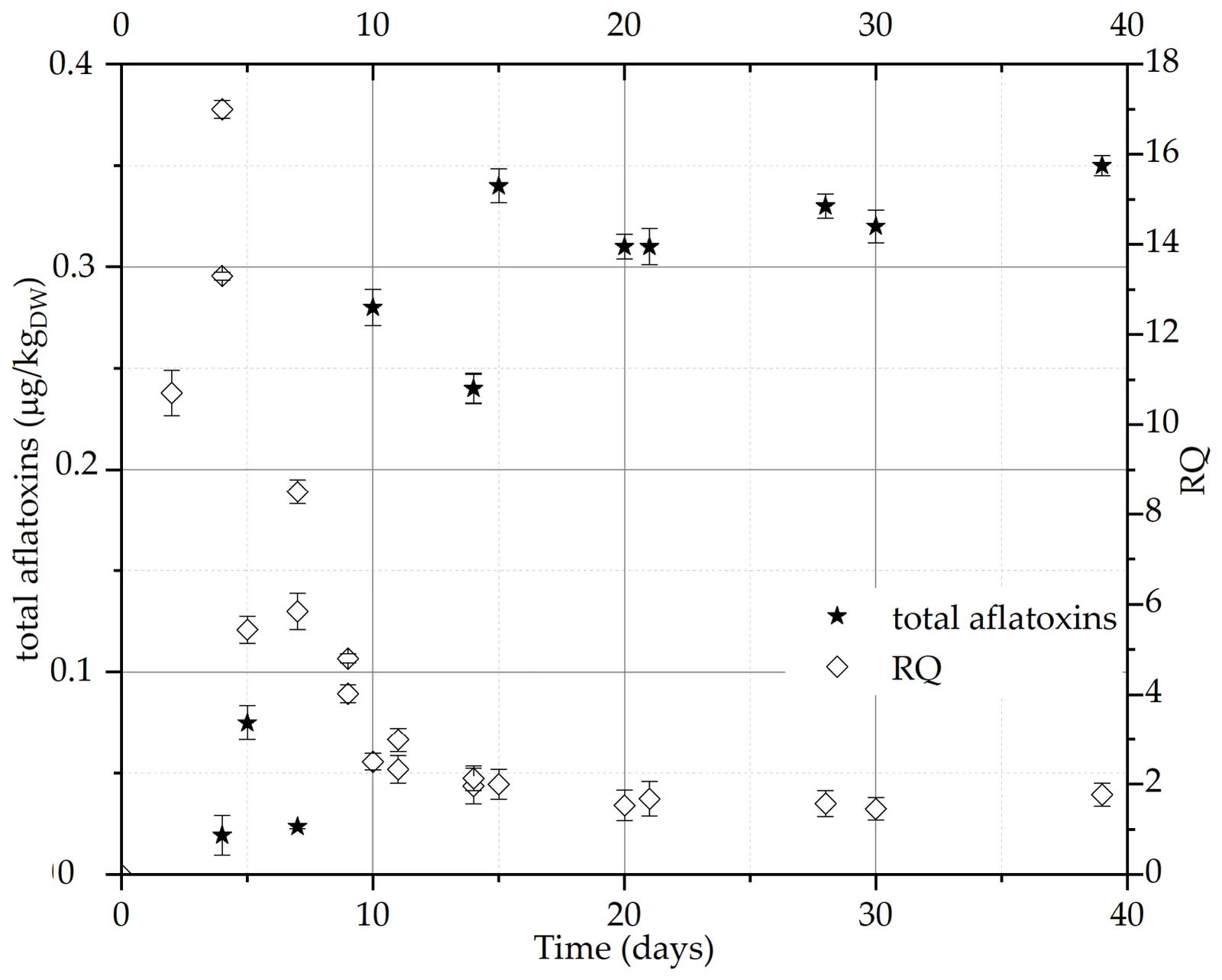
RQ and total aflatoxin values in relation to incubation time.

Tests in cone microcosm with respirometric sensor (Sm) were performed under the same experimental conditions as the previous test, using H hazelnuts, sampled from different batches (see Section 4.2.2). In these microcosm tests, in addition to CO_2_ evolution, the specific O_2_ uptake rate (RO_2_) was monitored throughout the incubation period, and the amount of consumed O_2_ and the RQ were calculated (see Table 4 and Figure 5). Starting from the 10th day of incubation, the average RQ value was of the same order of magnitude as that reported by [22] for an aflatoxigenic strain of *Aspergillus parasiticus* (1.3–3.2).

The graph in Figure 5 contains data from different experimental tests, carried out under the same experimental conditions using different batches of retail-sale H hazelnuts. As described below, four main clusters can be identified (see Table 4), based on the RQ values. Up to the 4th day of incubation (cluster 1), the RQ value was higher than 10 (between 10.7 and 13.3), while the mycotoxin concentration was approximately 0.02 μg/kg of hazelnuts, although with a relatively high margin of error. After that (cluster 2), the RQ decreases below 6 (7th day), whereas the mycotoxin concentration remained unchanged. At longer incubation times (10–14 days), cluster 3, the mycotoxin concentration (0.24–0.28 μg/kg) increased 10-fold, while the RQ value was halved (2.1–2.5). For lower RQ values (cluster 4), ranging from 1.53 to 1.9, a further increase in mycotoxin amount, 0.31–0.35 μg/kg, was observed, and this value remained approximately constant until the 40th day of incubation. Prolonging the test for a further 30 days, the RQ value remained constant (1.7), whereas the mycotoxin concentration increased to 0.51 μg/kg, confirming that mycotoxins were still produced during the stationary growth phase.

The data shown in Table 4 were used to establish a relationship between RQ values and aflatoxin concentrations. For this purpose, a preliminary data-smoothing step was required to properly account for measurement uncertainty and variability. As shown in Figure S3a and Figure S3b, respectively, aflatoxin concentration vs. time followed a logarithmic trend, whereas RQ vs. time followed an exponential trend. Therefore, it becomes possible to get a simple linear relationship between aflatoxins concentration and RQ that has been used in the model.

y = −0.0573x + 0.4235(1)

Equation (1) provides a reasonably accurate estimate of aflatoxin concentration, except for RQ values lower than 1 or higher than 7. In the former case, the aflatoxin concentration approaches an asymptotic value, whereas, in the latter case, the aflatoxin concentration is very low and therefore does not represent a significant concern.

### 2.7. Modeling for One-Step-Ahead and Long-Term Predictions

**Figure 6:**
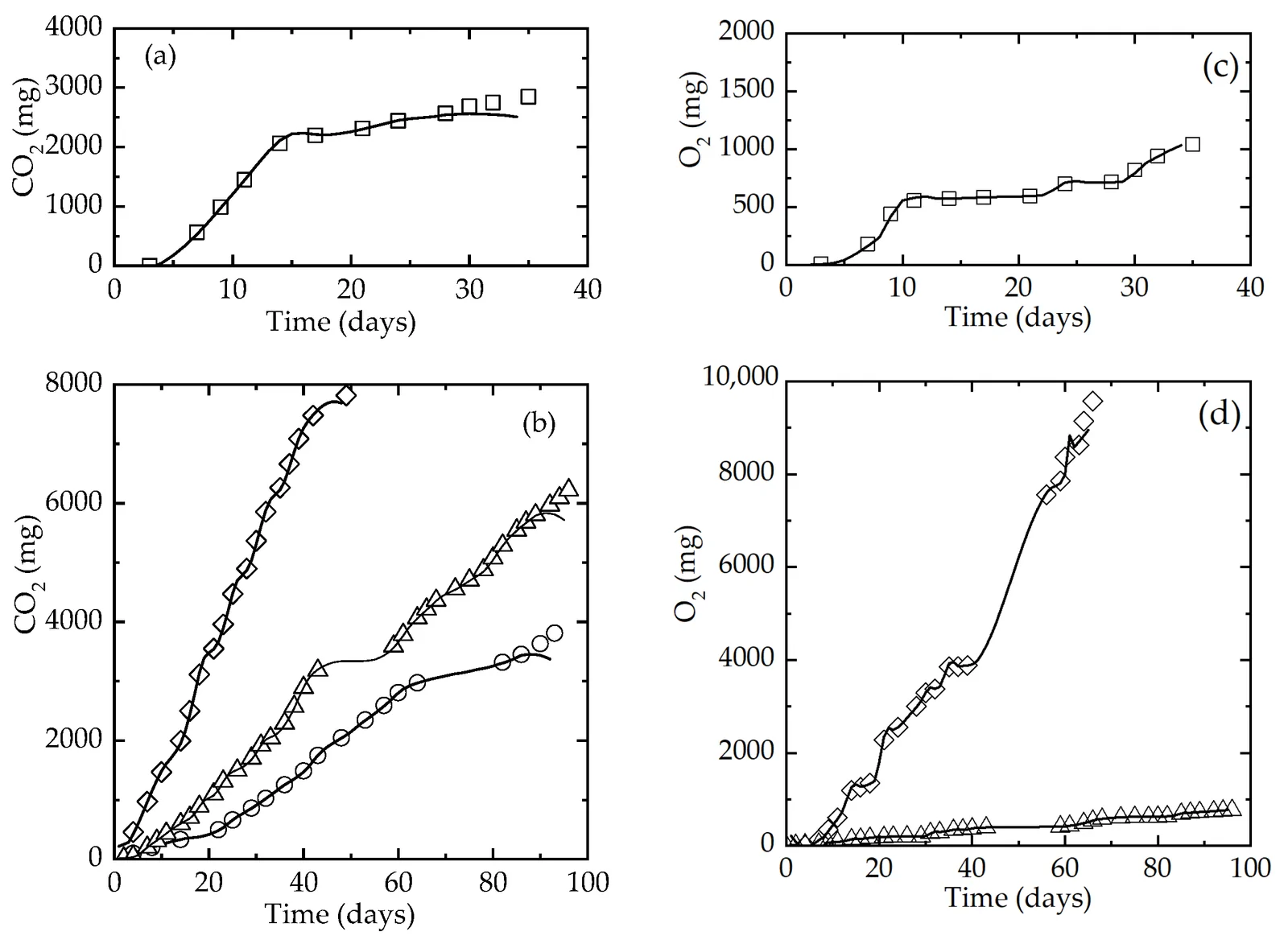
Comparison between values of produced CO_2_, (**a**,**b**), and between values of consumed O_2_, (**c**,**d**), in different considered tests. Symbol legend: squares are for D-W hazelnuts in Sm microcosm, Test (1); triangles are for H hazelnuts in Sm microcosm, Test (2); diamonds are for H hazelnuts in Sm microcosms, Test (3); circles are for H hazelnuts in Jm microcosm, Test (4). Symbols are experimentally measured values; lines are values estimated by the model for the one-step-ahead predictions.

The results obtained for CO_2_ when the model is used for one-step-ahead predictions are shown in Figure 6a,b: the obtained values of model parameters are a1 = 2.0002 and a2 = −1.0031; see Equation (7).

Figure 6a shows the results obtained in the learning step, in which the experimental results obtained in test (1) (D-W hazelnuts in Sm microcosm) were used to retrieve the model parameters. Figure 6b shows the results obtained when the model is used in tests (2), (3), and (4) (H hazelnuts in Sm microcosm, H hazelnuts in Sm microcosms, and H hazelnuts in Jm microcosm, respectively). In all cases, excellent agreement was observed between the measured and calculated CO_2_ values, with only a small difference at the end of the test.

The results obtained for O_2_ using one-step-ahead predictions are shown in Figure 6c,d. As in the previous case, test (1) was used for the learning step, whereas the others were used for validation; the values of model parameters were a1 = 1.8895 and a2 = −0.8874. The agreement was excellent both during the learning phase (Figure 6c) and during the validation phase (Figure 6d), demonstrating that the proposed model is capable of accurately estimating both CO_2_ and O_2_.

**Figure 7:**
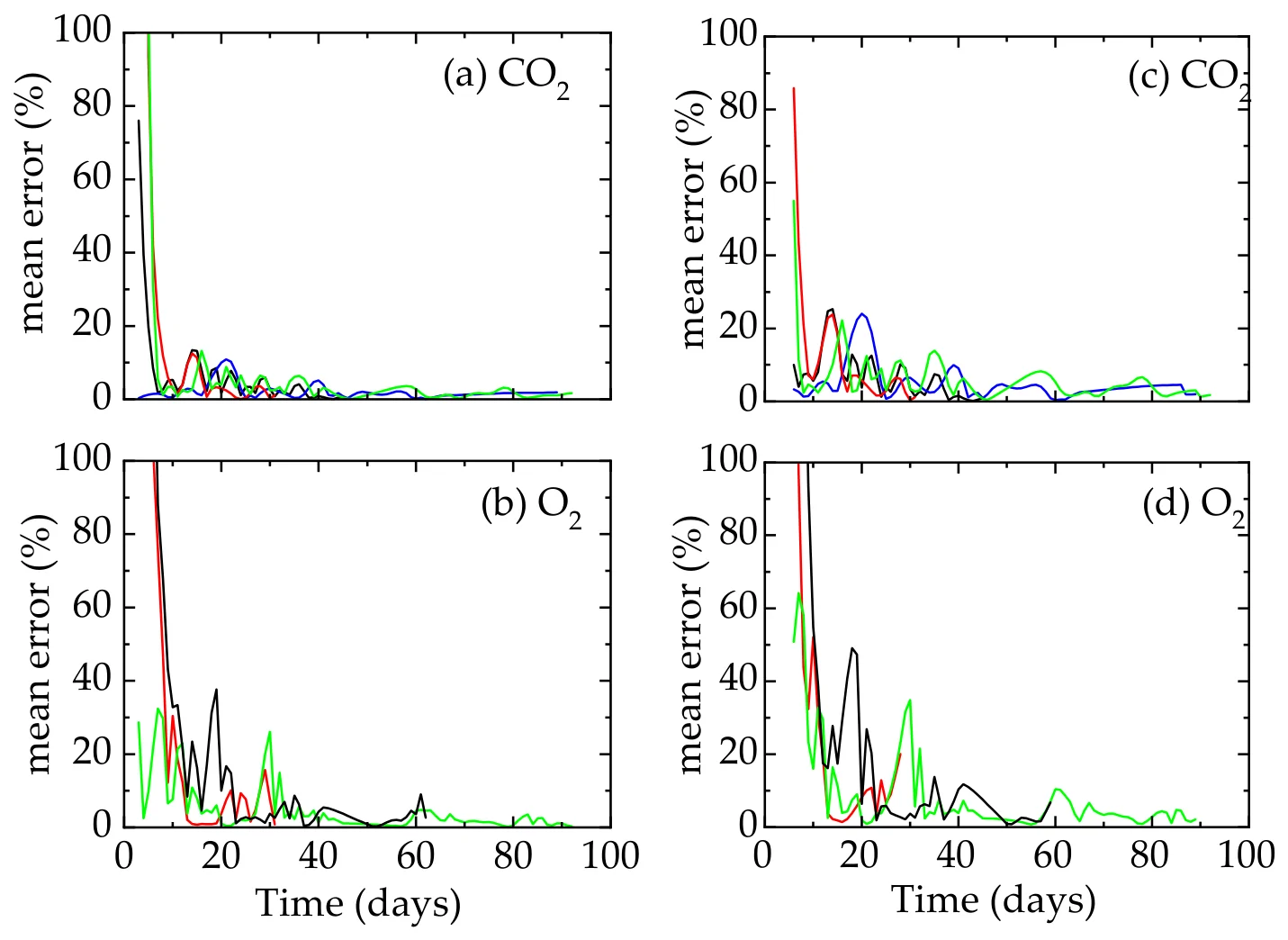
Mean error obtained applying the model for long-term predictions of the produced CO_2_ and consumed O_2_ amount. (**a**,**b**) refer to 3 days prediction; (**c**,**d**) refer to 7 days prediction. Color legend: D-W hazelnuts in Sm microcosm in red, Test (1); H hazelnuts in Sm microcosm in green, Test (2); H hazelnuts in Sm microcosms in black, Test (3); H hazelnuts in Jm microcosm in blue, Test (4).

As far as long-term predictions are concerned, Figure 7 shows the mean estimated error for CO_2_, (a), and O_2_, (b), considering a time interval of 3 days, while a time interval of 7 days is considered in Figure 7c,d. As expected, the 7 days’ time interval resulted in higher mean prediction errors, due to the propagation and the accumulation of errors associated with the iterative process. Nevertheless, except for the first 7–10 days from the onset of the test, the relative error on the estimation of both CO_2_ and O_2_ remained below 10% in most cases for the 3 days’ time interval. Slightly higher errors were observed for CO_2_ and O_2_ for the 7 days’ time interval.

It has to be highlighted that the higher uncertainty in the estimate, observed at the beginning of the test, is not a significant concern since the amount of mycotoxin produced during this period is very low. Consequently, the larger prediction errors occurring at this stage do not represent a relevant risk from a food safety perspective. Therefore, the CO_2_ and O_2_ estimates, obtained in the first 10 days, should not be used for decisions regarding the presence of contaminants.

**Figure 8:**
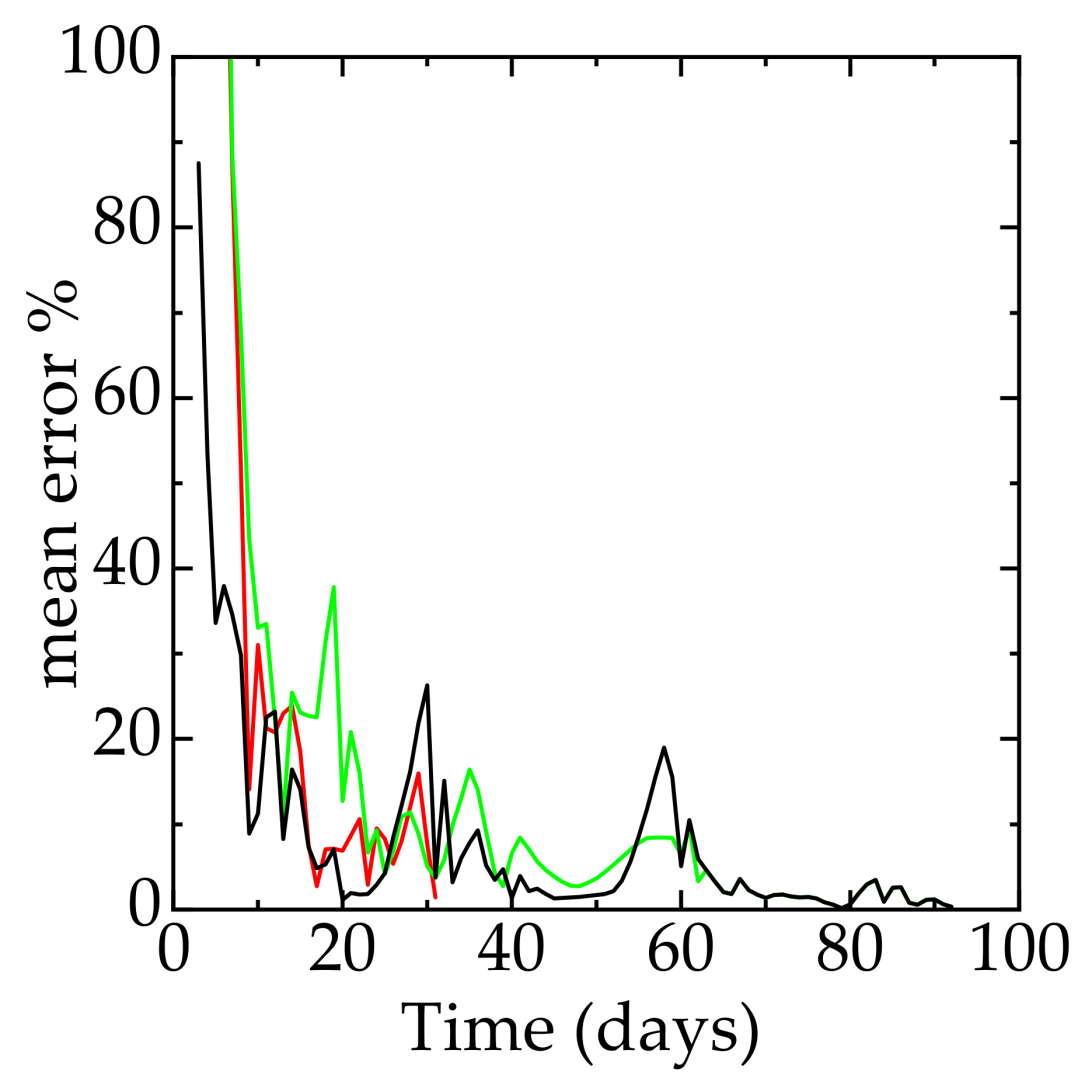
Estimated mean error of RQ obtained applying the model for long-term (3 days) predictions of the produced CO_2_ and consumed O_2_. Color legend: D-W hazelnuts in Sm microcosm in red, Test (1); H hazelnuts in Sm microcosm in green, Test (2); H hazelnuts in Sm microcosms in black, Test (3).

As detailed in Section 4.7, since the experimental CO_2_ and O_2_ values are used to estimate the RQ, the RQ uncertainty can be calculated from the uncertainty of CO_2_ and O_2_. Figure 8 shows the calculated mean error on the prediction for the RQ over a time interval of 3 days. Except for the first 10 days, the mean error is generally lower than 10% and can therefore be considered acceptable for in-line applications.

The RQ values were used to evaluate the mean error in the estimation of mycotoxin concentrations, as described in Section 4.7. In this case, it should be highlighted that A = 0.4235 and B = 0.0573 and that, except for the first 10 days, the RQ values ranged from 1 to 4 in all the tests considered. Since a linear relationship exist between the amount of mycotoxins and the RQ, the absolute error on the amount of mycotoxins is equal to the absolute error on the RQ multiplied by 0.0573. Consequently, even a relevant error in the RQ estimate has only a limited impact on the estimated mycotoxin amount. As far as the relative error is concerned, because the RQ is multiplied by 0.0573 and the product is significantly lower than 0.4235, the relative error on the amount of mycotoxins is lower than the relative error in the RQ. Therefore, the proposed algorithm is suitable for the in-line estimation of the amount of produced mycotoxins.

### 2.8. Advantages and Limitations of the Proposed Soft-Sensors

The proposed soft-sensors allow both one-step-ahead and long-term predictions of the CO_2_ produced and O_2_ consumed and, consequently, the value of RQ. This is achieved by combining measured CO_2_ and O_2_ data with an autoregressive model trained on a limited number of previous tests (as demonstrated in this study, even a single test may be sufficient). From the RQ value, the amount of produced aflatoxins may be predicted, provided that the relationship between the RQ and aflatoxin content is known. In this work, this relationship was established through previous experimental investigations.

This approach offers several advantages over currently available methods for monitoring hazelnuts storage conditions:

- Cost-effective and rapid response: the system is based on low-cost sensors, avoiding specialized laboratory equipment and reducing the long analysis times typically associated with traditional analytical methods.
- Non-destructive and in-line monitoring: unlike traditional analysis (such as HPLC or ELISA), which requires grinded samples and may involve sample destruction, the proposed system may be used for in-line monitoring of both whole and shelled hazelnuts, without compromising the product.
- High predictive accuracy: the inverse linear relationship found in this study between RQ and aflatoxin content ensures that small errors in the estimation of gas concentration reflects into minimal variations in estimated mycotoxins.
- Proactive hazard mitigation: the proposed sensor system has to be considered as an early warning tool in storage facilities, alerting operators to the potential presence of mycotoxigenic fungi before aflatoxin concentration reaches critical threshold values.

Nevertheless, the proposed system also presents some limitations:

- Lag in model stability: the autoregressive model requires data for model training and an initial calibration period. Approximately 7–10 days of data collection are required to obtain a prediction error lower than 10%.
- Indirect measurement of contamination: because the sensor monitors cellular respiration, any microorganisms capable of consuming oxygen and producing carbon dioxide, including non-aflatoxigenic species, may influence the calculated RQ values.
- Effects of temperature and moisture: temperature and moisture content, which are known to influence mycotoxins production, can be easily monitored. Therefore, they could be included as exogenous inputs into the autoregressive model. This aspect will be investigated in future studies.

## 3. Conclusions

In the present work, a respirometric sensor system, usually applied in soil microcosm, was adopted to monitor microbial growth on hazelnuts, which was subsequently correlated to mycotoxin production. The experimental data were used to develop a simple autoregressive model, and an excellent agreement was obtained between the measured and estimated values. The present work should be considered as a feasibility study and requires further development to become a practical tool for the post-harvest management of hazelnuts.

## 4. Materials and Methods

### 4.1. Hazelnut Characterization: Morphological and Chemical/Physical

In the present work, various types of hazelnuts were used: brown marmorated stink bug (*Halyomorpha halys* Stal) damaged, both whole and broken, from the wholesale trade (La Gentile s.r.l., Cortemilia, CN), and, whole, healthy hazelnuts, from the retail sale, with or without the shell (Table 5).

**Figure 9:**
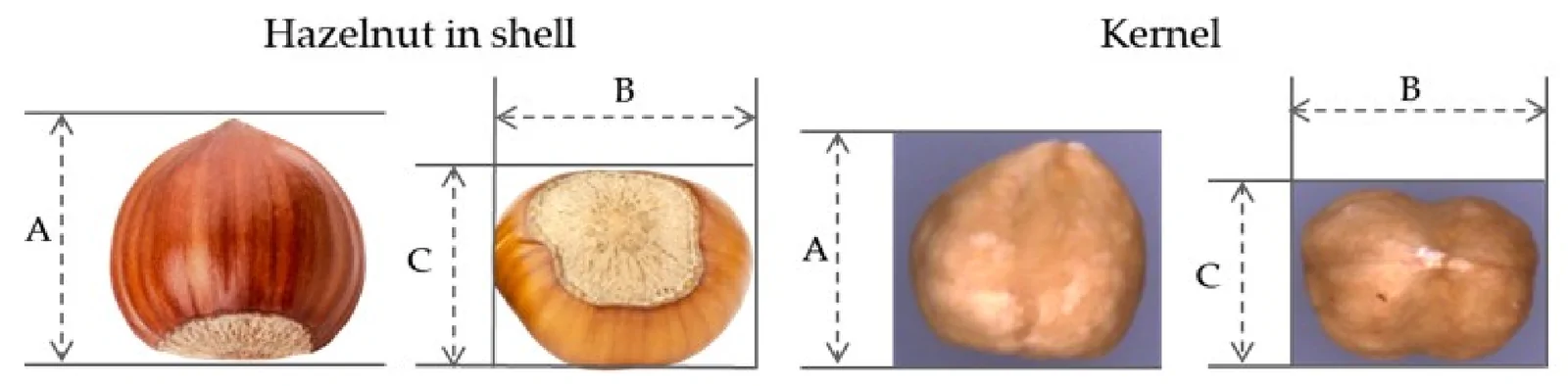
Hazelnuts in shell and kernels measured dimensions (A, B, C).

The morphological characteristics of each type of hazelnut were evaluated considering 25 samples, randomly collected. As shown in Figure 9, dimensions (length, A, width, B, and thickness, C, in mm) were manually measured using a caliper (precision of 0.01 mm).

The average diameter size, Dp, and sphericity, Ψ, of the kernels were calculated by the geometric mean of the three dimensions, A, B, and C, according to Equations (2) and (3), respectively [2,22,23].

(2) $$Dp={\left(A\cdot B\cdot C\right)}^{\frac{1}{3}}$$

(3) $$\Psi =\frac{Dp}{A}$$

The weight (g) was assessed with a digital scale (precision of 0.001 g; SCALE-TEC, Vadodara, Gujarati, India). Moisture % was measured by means of a thermobalance (MB120, OHAUS Europe GmbH Heuwinkelstrasse 3, Nänikon, Switzerland) at 80 °C, operating at a drying rate of 1 mg/90 s. As regards pH assessment, hazelnuts were mixed with distilled water at a ratio of 1:2.5 (w_g_/v_mL_), at 25 °C, and under continuous stirring (C-MAG HS 4, IKA, Staufen, Germany) at 1000 rpm for 1 h; this suspension was left to settle for about 30 min and the pH of the water supernatant was evaluated with a benchtop pH meter (pH730, inoLab, WTW, Weilheim, Germany).

### 4.2. Microcosm Set-Up

#### 4.2.1. Microcosm Tests in Cylindric Microcosms

**Figure 10:**
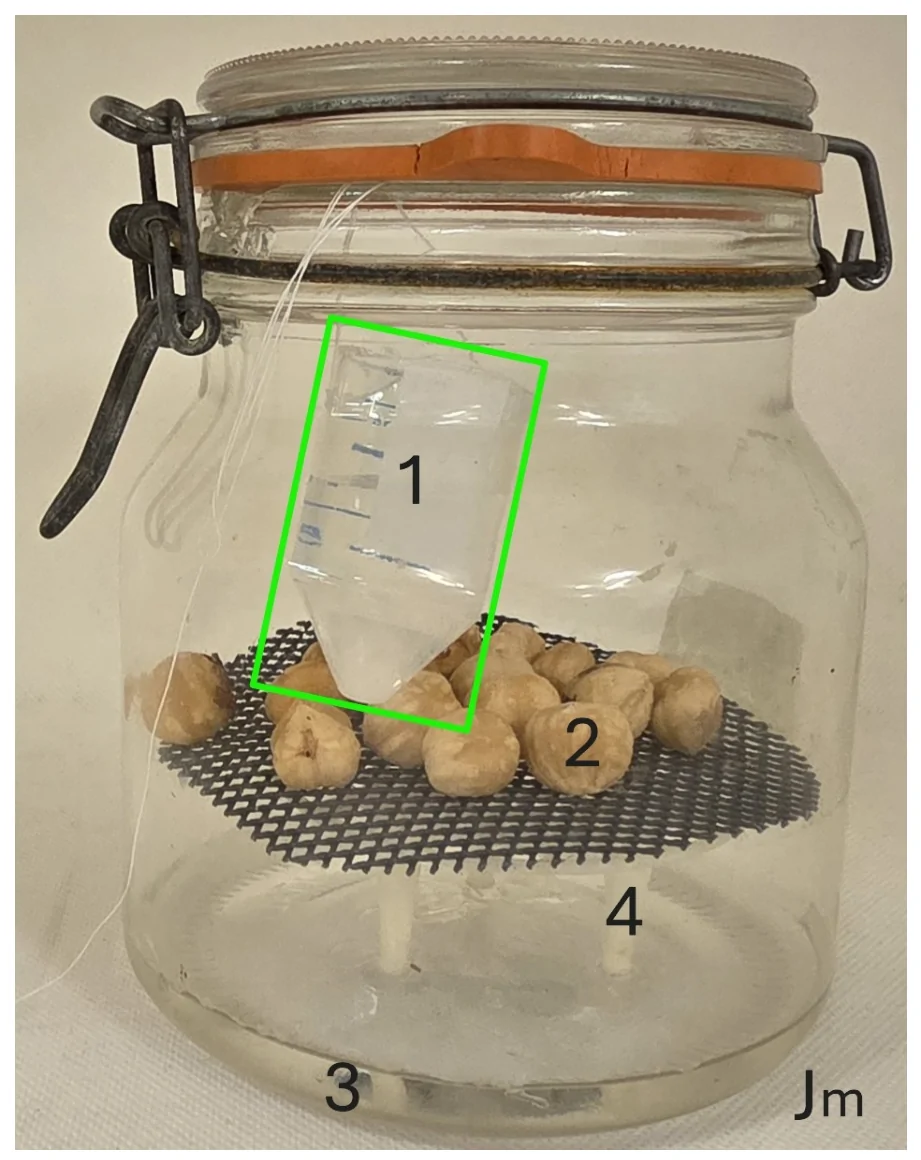
Cylindric jars: Jm microcosms. (1) Tube for NaOH solution; (2) hazelnut monolayer on the plastic grid; (3) water; (4) grid support.

First microcosm tests were carried out in cylindric glass jars with a free internal volume of 1 L and with a closure secured by a sealing gasket (microcosms Jm, see Figure 10).

Damaged BMSB hazelnuts (D-W, D-B, and a mixture of these two, D-WB), and healthy ones, with or without shell (H-S and H, respectively), were used. An amount corresponding to 35 g_DW_ (dry weight) was arranged in monolayer above a plastic net (4 mm net) and microcosm was maintained at 40% of relative humidity (R.H.) by adding 20 mL of distilled water at the bottom of the jar. A polypropylene tube (20 mL capacity), filled with a NaOH solution to capture the produced CO_2_, was hung at the neck of the jar. All the microcosm components, shown in Figure 10, were sterilized in autoclave (121 °C, 2 atm, for 20 min); in biotic microcosms nuts were used as is, while, in abiotic ones, they were sterilized together with the jar.

Jars were incubated in the dark at 25 °C; biotic and abiotic microcosms were periodically aerated, every 3 or 4 days, maintaining the cup open for 15 min (the operation was carried out under sterile conditions). Each time microcosms were opened, the NaOH solution was collected and substituted with a new one, before the jar closure [24]. At the beginning and at the end of the incubation period, 23 days, total aflatoxins were extracted from each type of hazelnut and quantified (see Section 4.5).

#### 4.2.2. Microcosm Tests in Cone Microcosms

**Figure 11:**
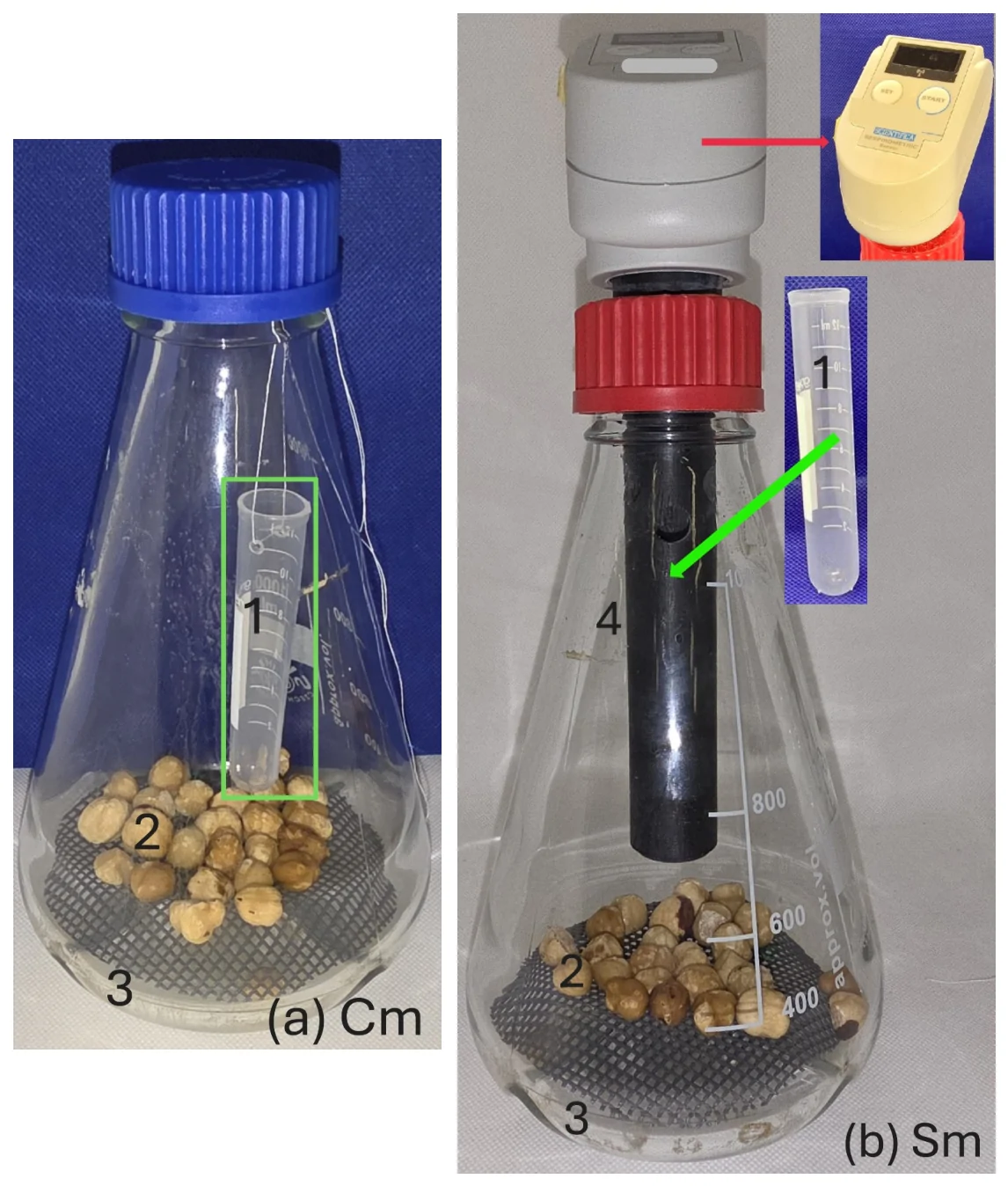
Cone bottles: Cm (**a**) and Sm (**b**) microcosms. (1) Tube for NaOH solution; (2) hazelnut monolayer on the plastic grid; (3) water; (4) holding tube. In (**b**) the respirometric sensor details are shown with a red arrow.

In addition to the cylindric jars (Jm microcosms), microcosms were also arranged in cone bottles (volume 1.025 L, see Figure 11), closed with a screw cap (Cm microcosms) or a cap equipped with a respirometric sensor (VELP RESPIROMETRIC sensor, VELP Scientifica Srl, Usmate, Italy), which allows the O_2_ consumption rate (Sm microcosms) to be evaluated. Also in cone microcosms, a monolayer of 35 g_DW_ of healthy hazelnuts (H) was arranged on the plastic net at 3 cm from the bottom, and 20 mL of distilled water was added to maintain the 40% relative humidity, R.H. The polypropylene tube (20 mL capacity), containing the NaOH solution, was hung at the neck of the bottle in Cm microcosms, while, in Sm ones, it was arranged in the holding tube connected to the respirometric sensor; NaOH solution was changed at each microcosm opening. As described for Jm microcosms, all elements of Cm and Sm ones underwent autoclave sterilization, with the sole exception of the hazelnuts of biotic tests; also, incubation conditions and the aeration procedure were the same. As regards aeration frequency, different time intervals were tested, from 1 day to a maximum of 25, depending on the test, as described in Section 2 (see Section 2.4, Section 2.5 and Section 2.6). Total aflatoxins were extracted from samples collected at the beginning, during the incubation period and at its end, as detailed in Section 2.6.

### 4.3. Microbiological Tests

The evaluation of the biomass naturally present on the kernel surface was carried out on H hazelnuts, sampled before and during microcosm incubation. Operating in sterile conditions, nuts were mixed with 0.85% physiological solution in a ratio 1:2 (w_g_/v_mL_) and maintained in agitation at 125 rpm for 30 min at 25 °C. Then, 100 µL of the supernatant were spread on Malt Extract Agar, MEA (D(+)glucose 20 g/L, malt extract 20 g/L, peptone 2 g/L, and 2% agar), plates prepared at pH 6 with chloramphenicol (0.01%) added or not, which makes the medium selective for yeasts and fungi. Inoculated dishes were incubated at 25 °C and microbial growth was visually inspected after 144 h.

### 4.4. Respirometric Analysis

The produced CO_2_, captured in the 3 M NaOH solution, dispensed into the sterile polypropylene tubes (see Figure 10 and Figure 11), was measured according to the method described in [24]. CO_2_ was quantified using an acid–base titration with HCl 1.5 N, BaCl_2_ 1 M as the precipitating agent, and a 1% phenolphthalein solution in 80% ethanol, as a color indicator, and the amount was calculated as follows; see Equation (4):

(4) $${CO}_{2}\;\left(mg\right)=\;\left(V_{0}\;-\;V\right)\cdot f$$

V_0_ is the HCl volume necessary to titrate the sterile abiotic controls, V is the volume required for the biotic samples, and f is the conversion factor equal to 22× M, where M is the HCl solution molarity.

The consumed O_2_ was monitored with a commercial sensor, which registers the Biological Oxygen Demand (BOD) values and the pressure variation in the closed microcosm; see Figure 11b. The system allows the collected data to be directly saves: the sensor is managed by the RESPIROSoft™ software (VELP Scientifica Srl, Usmate, Italy) and provides results as pressure values (mbar). The wireless connection is guaranteed by a DataBoxTM (VELP Scientifica Srl, Usmate, Italy) and data are collected in real time. The specific rate of oxygen consumption, RO_2_ (mg/g·h), was calculated according to Equation (5).

(5) RO2=32,000 Vfg · ∆Pt·T·R·msd

In Equation (5), 32,000 is the oxygen molar mass (mg/mol), V_fg_ is the gas free volume (L), R is the gas constant (83.14 hPa·L/mol·K), t is the time (h), T is the temperature (K), m_sd_ is the hazelnut dry mass, and ΔP is the pressure difference (hPa) in the time interval of interest.

Knowing the CO_2_ and O_2_ amount, the RQ can be calculated from the ratio of the two values.

### 4.5. Total Aflatoxin Quantification

Aflatoxins were extracted with 70% methanol aqueous solution, from hazelnuts maintained at −20 °C, according to the procedure provided for the commercial kit used for aflatoxin determination (RIDASCREEN^®^ Aflatoxin Total kit, REF R4701, R-biopharm, Darmstadt, Germany); the detection limit is <0.05 µg/L of extract. Before the extraction, a washing step was carried out to remove the biomass present on the surface: an amount of 10 ± 0.5 g of thawed nuts was mixed with distilled water (ratio 1:4, g:mL), kept in agitation for 30 min at room temperature, and centrifuged at 4000 rpm for 12 min at +4 °C. Washed hazelnuts were then dried at 60 °C for 24 h and milled (JANKE & KUNKEL, IKA A10, Staufen, Germany) till a homogeneous product was obtained. Mycotoxins were extracted with 70% methanol added to milled nuts in a ratio 1:5 (g:mL), maintained in agitation at 150 rpm for 10 min at room 25 °C, and separated by filtration with qualitative filter paper Whatman (N.1) (Whatman International Ltd., Maidstone, UK).

### 4.6. Autoregressive Modeling

The aim of this part of the study was to develop a mathematical model capable of predicting CO_2_ evolution and O_2_ consumption during hazelnut storage. These values were then used to calculate the RQ and, from this, the produced mycotoxins amount.

Although the experimental results showed a simple relationship between the RQ and mycotoxins amount, enabling effective in-line monitoring, the possibility of estimating the evolution of the system is of great importance for the early identification of potentially hazardous conditions. This objective requires the use of a mathematical model.

The use of first-principle models is not feasible in this case. In addition to the difficulty of properly accounting for the heat and mass transfer phenomena involved, as well as the kinetics of the reactions occurring, it would d be extremely challenging, if not impossible, to set-up suitable experiments to get all the parameters involved. Therefore, we exploit the possibility of using simple black-box models, trained with experimental data.

Within this framework, a simple auto-regressive model was initially selected, while retaining the possibility of using more complex models if the accuracy obtained proved insufficient. In an auto-regressive model the current value of a variable (y_t_) is expressed as a linear combination of its previous values (y_t−1_, y_t−2_, y_t−3_ …); see Equation (6):

(6) $$y_{t}=\sum_{i}a_{i}\;y_{t-i}\;$$

In a first attempt, which was later found to be capable of providing results with an acceptable degree of accuracy, only two past measured values of the variable y were considered, with a constant time interval equal to one day, i.e., the value of the variable y at time t − 1 (y_t−1_) corresponds to the variable one day before the value obtained at time t (y_t_), and so on.

Two models were developed; in the first, the variable y corresponds to the amount of CO_2_, while, in the other, to the amount of O_2_. The values of the parameters a_i_, the coefficients of the linear combination, are retrieved looking for the best fit between calculated and measured values of the target variable (CO_2_ and O_2_) in a single set of data.

The resulting model was used in two different ways:

(1) One-step ahead prediction: the available (measured) values of y (at times t and t − 1) are used to estimate the value of this variable at time t + 1, i.e., one day later, see Equation (7):

(7) $$y_{t+1,estimated}=\;a_{1\;}y_{t,measured\;}+\;a_{2\;}y_{t-1\;measured\;}$$

(2) Long-term prediction: in this case, not only y_t+1_ is estimated but also future values. As these measurements are unavailable, the model operates recursively, feeding its own past forecasts back into the model Equations (8) and (9):

(8) $$y_{t+2,estimated}=\;a_{1\;}y_{t+1,estimated\;}+\;a_{2\;}y_{t,measured\;}$$

(9) $$y_{t+3,estimated}=a_{1\;}y_{t+2,estimated\;}+a_{2\;}y_{t+1,estimated}$$

The accuracy of the model used for one-step-ahead predictions was evaluated through a graphical comparison between estimated and measured values (CO_2_ or O_2_, depending on the model); as will be shown in the following, the excellent fitting made the use of more complex models unnecessary, thus avoiding the need for additional performance parameters.

The accuracy of the model for long-term predictions was assessed by evaluating the mean value of the difference between calculated and measured values (of CO_2_ or O_2_, depending on the model) over a given time interval. In this study, 3 days and 7 days will be considered. Obviously, the error is expected to increase in case of long-time horizons as the iterative process leads to the propagation and accumulation of modeling errors [19].

**Figure 12:**
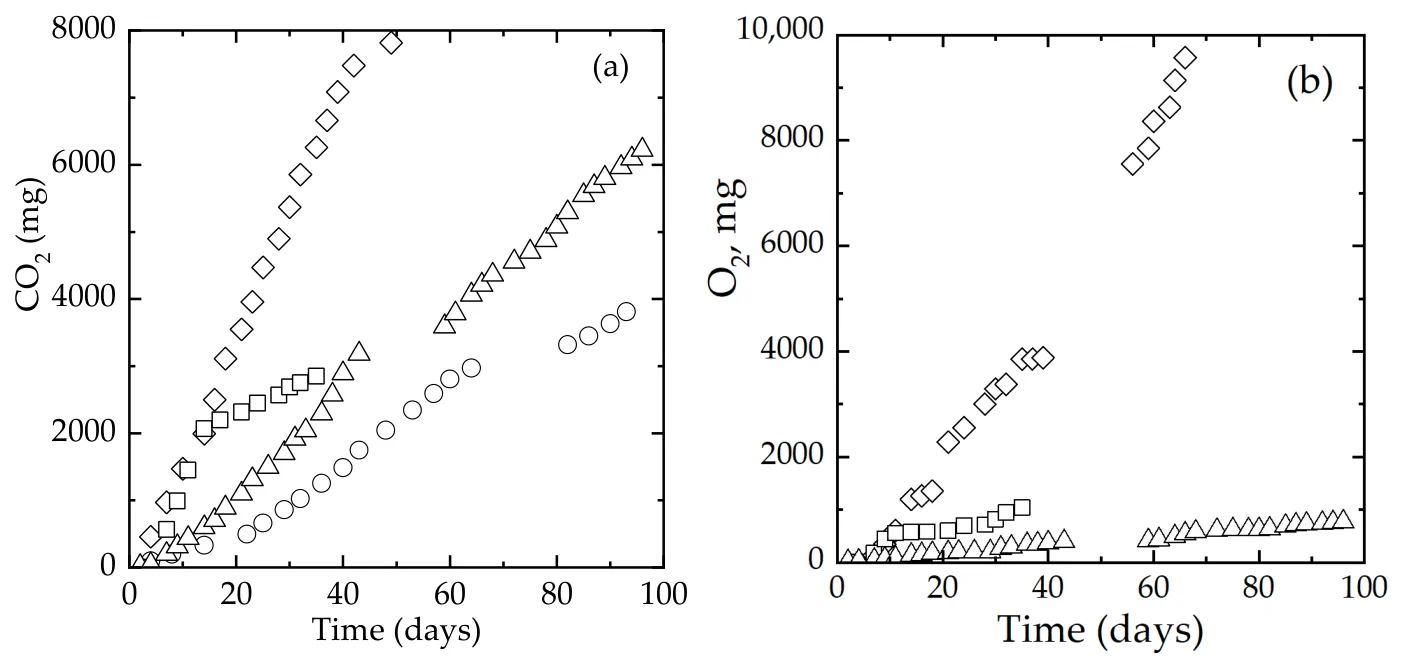
Time evolution of produced CO_2_ (**a**) and consumed O_2_ (**b**) values used in the learning and validation steps of the model development. Symbol legend: squares are for D-W hazelnuts in Sm microcosm, Test (1); triangles are for H hazelnuts in Sm microcosm, Test (2); diamonds are for H hazelnuts in Sm microcosms, Test (3); circles are for H hazelnuts in Jm microcosm, Test (4).

As far as the model for CO_2_ evaluation is concerned, four tests were considered, see Figure 12: (1) D-W hazelnuts in Sm microcosms, (2) H hazelnuts in Sm microcosm, (3) H hazelnuts in Sm microcosms, and (4) H hazelnuts in Jm microcosm. The CO_2_ values obtained in these tests are shown in Figure 12a. Test (1) was used in the learning phase, i.e., to estimate the values of model parameters a_i_, while the others (i.e., (2), (3), and (4)) were used for model validation.

As far as the model for O_2_ evaluation is concerned, tests (1), (2) and (3), where the amount of O_2_ was measured, were considered. The O_2_ values obtained in these tests are shown in Figure 12b. Also in this case, test (1) was used in the learning phase, i.e., to estimate the values of model parameters ai, while the others were used for model validation. Once CO_2_ and O_2_ had been calculated, the values were used to evaluate the RQ and, from this, the amount of mycotoxins produced.

### 4.7. Uncertainty Evaluation of Model Estimates

For both CO_2_ and O_2_, model-estimated values must be compared to experimentally measured ones to obtain the error of the model. The relative error may be easily calculated dividing the error by the real value. As the RQ is calculated from the ratio of CO_2_ and O_2_ amount, being $\varepsilon_{CO2}$ and $\varepsilon_{O2}$ the relative error on the value of CO_2_ and O_2_, respectively, the relative error on the value of the RQ was calculated using Equation (10):

(10) $$\varepsilon_{QR}=\;\sqrt[{}]{{\left(\varepsilon_{CO2}\right)}^{2}+\;{\left(\varepsilon_{O2}\right)}^{2}}\;$$

Finally, considering that Equation (10) relating the amount of mycotoxins (y_Afl_) to the respirometry quotient (y_RQ_) is:

(11) $$y_{Afl\;}=A-By_{RQ}$$

the relative error on y_Afl_ is seen in Equation (12):

(12) $$\varepsilon_{Afl\;}=\;\varepsilon_{RQ\;}\frac{\left|{By}_{RQ}\right|}{\left|A-{By}_{RQ}\right|}$$

### 4.8. Statistical Analysis

The reported data are means of three biological replicates with their standard deviation (SD), shown in graphs as error bars. Differences between two given groups were tested for statistical significance using Student’s *t*-test (*p* < 0.05), while those between more than two groups were evaluated by one-way analysis of variance (ANOVA) followed by the Bonferroni test. Differences with *p* < 0.05 are statistically significant; “ns” is reported for *p* > 0.05, “*” for *p* < 0.05, “**” for *p* < 0.01, “***” for *p* < 0.001, and “****” for *p* < 0.0001.

## Figures and Tables

**Table 1:** Hazelnut characterization.

	A (mm)	B (mm)	C (mm)	Dp (mm)	Ψ	Weight (g)	Moisture (%)	pH
**D-B**	12.0 ± 2.1	11.3 ± 1.8	5.3 ± 1.0	8.9 ± 1.3	0.74 ± 0.12	0.54 ± 0.12	8.0 ± 1.38	6.1 ± 0.1
**D-W**	13.0 ± 1.5	12.0 ± 1.0	10.0 ± 1.2	11.6 ± 1.0	0.90 ± 0.09	1.10 ± 0.2	7.5 ± 0.3	6.1 ± 0.1
**H**	12.7 ± 1.1	12.4 ± 0.8	11.3 ± 1.2	12.1 ± 0.9	0.95 ± 0.09	0.95 ± 0.18	6.9 ± 0.3	6.2 ± 0.1
**H-S**	17.0 ± 1.8	16.3 ± 1.2	15.3 ± 1.0	n.d.	n.d.	2.25 ± 0.1	10.8 ± 0.2	6.0 ± 0.1

n.d. is for “not determined”.

**Table 2:** CO_2_ produced in the presence of different hazelnut type (mg/g_DW_).

Time (Day)	H-S	H	D-W	D-B	D-WB
4	0.0 ± 0	0.0 ± 0	1.2 ± 0.2	1.1 ± 0.1	1.4 ± 0.1
7	2.9 ± 0.68	3.8 ± 0.71	9.0 ± 0.74	9.0 ± 1.38	9.5 ± 1.53
10	5.7 ± 0.78	7.6 ± 0.81	19.9 ± 0.97	20.5 ± 1.39	16.2 ± 1.37
14	9.5 ± 0.81	12.4 ± 0.84	27.9 ± 1.18	33.4 ± 1.91	25.7 ± 1.26
17	13.3 ± 0.92	19.0 ± 0.94	38.9 ± 1.29	41.8 ± 1.78	40.0 ± 1.47
20	17.1 ± 1.0	25.7 ± 1.29	51.2 ± 1.38	54.9 ± 1.71	50.4 ± 1.21
23	21.9 ± 0.68 (0.067) *	37.1 ± 1.75 (0.16) *	62.6 ± 1.56 (0.16) *	68.6 ± 1.97 (0.19) *	59.0 ± 2.14 (0.12) *

* Specific CO_2_ production rate (from 20th to 23rd day), mg/g_DW_h.

**Table 3:** Cumulated CO_2_ (mg/g_DW_) and specific CO_2_ production rate (mg/g_DW_∙h) in microcosms with different geometry. Biomass development at the 11th day is also shown.

Time (Day)	Jm CO_2_		Cm CO_2_		Sm CO_2_	
	Cumulate mg/g_DW_	Production Rate mg/g_DW_∙h	Cumulate mg/g_DW_	Production Rate mg/g_DW_∙h	Cumulate mg/g_DW_	Production Rate mg/g_DW_∙h
2	20.6 ± 0.9	0.43 ± 0.02	20.9 ± 0.9	0.43 ± 0.02	20.7 ± 0.9	0.43 ± 0.02
4	38.5 ± 0.5	0.37 ± 0.01	39.1 ± 0.7	0.38 ± 0.01	37.0 ± 0.5	0.34 ± 0.01
6	52.8 ± 0.3	0.30 ± 0.01	50.5 ± 0.4	0.24 ± 0.01	51.6 ± 0.3	0.31 ± 0.01
9	69.4 ± 0.2	0.23 ± 0.01	67.7 ± 0.2	0.24 ± 0.01	66.7 ± 0.2	0.22 ± 0.01
11	82.4 ± 0.2	0.27 ± 0.01	81.8 ± 0.2	0.30 ± 0.01	81.0 ± 0.2	0.30 ± 0.01
	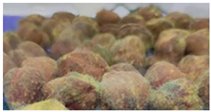		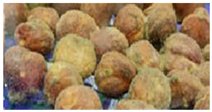		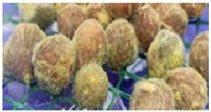	

**Table 4:** Consumed O_2_, produced CO_2_, and RQ values for H hazelnuts in Sm microcosm. The gray-scale sections (2–4, 5–7, 10–14, and 15–28 days) were compared for statistical significance of total aflatoxin concentration (ug/kg_DW_). Aflatoxin concentration for H hazelnuts before incubation is 0.019 ± 0.0098 µg/kg_DW_; that at the 40th day of incubation is 0.35 ± 0.005.

Time (Day)	Consumed O_2_ (mg)	Produced CO_2_ (mg)	RQ (CO_2_/O_2_)	Aflatoxins (μg/kg_DW_)	Cluster			
2	29.2 ± 1.35	312 ± 14.5	10.7	0.02 ± 0.012	1	*		
4	46.4 ± 1.3	617 ± 4.2	13.3	0.02 ± 0.0085				
5	136 ± 7.4	740 ± 40.8	5.44	0.04 ± 0.033	2		***	
7	178 ± 12.0	1038 ± 70.0	5.84	0.02 ± 0.001				
10	562 ± 38.1	1450 ± 98	2.5	0.28 ± 0.009	3			
11	882 ± 56.5	2057 ± 155.6	2.33	0.23 ± 0.0055				***
14	1140 ± 212.6	2370 ± 320.2	2.1	0.24 ± 0.007				
14	1170 ± 198.1	2505 ± 300.5	2.13	0.24 ± 0.008				
15	1100 ± 181.0	2200 ± 363	1.9	0.34 ± 0.0083	4			
20	1500 ± 310	2300 ± 310.5	1.53	0.31 ± 0.0061				
21	2303 ± 420	3878 ± 677.3	1.68	0.32 ± 0.008				
28	3053 ± 523.3	4793 ± 312	1.57	0.33 ± 0.0065				

For statistical significance: “*” is for *p* < 0.05, “***” is for *p* < 0.001.

**Table 5:** Hazelnut types.

Source	Hazelnut Characteristics			Acronyms
Wholesale	Damaged (BMSB)	Whole	Shelled	D-W
Wholesale	Damaged (BMSB)	Broken	Shelled	D-B
Retail sale	Healthy	Whole	Shelled	H
Retail sale	Healthy	Whole	With shell	H-S

## Data Availability

The original contributions presented in this study are included in the article/Supplementary Materials. Further inquiries can be directed to the corresponding author.

## Supplementary Materials

The following supporting information can be downloaded at: https://www.mdpi.com/article/10.3390/toxins18090385/s1, Figure S1. Colonies growth on MEA (a) and MEA added with chloramphenicol (b). Figure S2. Cumulated CO_2_ (mg/g_DW_) produced in microcosms with different geometry (cylindric Jm, and cone, Cm and Sm) with H hazelnuts. For all the incubation times: *p* > 0.05. Figure S3. Total aflatoxin (a) and RQ values vs. time (b).

## Abbreviations

The following abbreviations are used in this manuscript:

ANOVA	One-way analysis of variance
BMSB	brown marmorated stink bug
BOD	Biological Oxygen Demand
Cm	Cone bottles microcosms with screw cap
D-B	Broken damaged hazelnuts
Dp	average diameter size
DW	Dry weight
D-W	Whole damaged hazelnuts
D-WB	Whole and broken damaged hazelnuts
H	healthy hazelnuts without shell
H-S	healthy hazelnuts with shell
Jm	microcosm in cylindric glass jars
MEA	Malt extract agar
msd	hazelnut dry mass
R	gas constant
R.H.	relative humidity
RO_2_	specific O_2_ consumption rate
RQ	Respirometric quotient
Sm	Cone bottles microcosms with respirometric sensor cap
t	time
T	temperature
Vfg	gas free volume
ΔP	pressure difference
Ψ	sphericity
