# Supplementary material for: On the Application of Respirometric System on Hazelnut Samples to Predict Aflatoxin Production
Source: Toxins (Basel). 2026 Sep 6;18(9):385. doi: 10.3390/toxins18090385 (PMC13611684; doi:10.3390/toxins18090385)
Supplement: Supplementary file 1 [file toxins-18-00385-s001.zip › toxins-4458499-supplementary.pdf]

Supplementary materials: On the Application of Respirometric System on Hazelnut Samples to Predict Aflatoxin Production

Francesca Bosco, Chiara Mollea, Gabriele Baldissone, Micaela Demichela and Davide Fissore

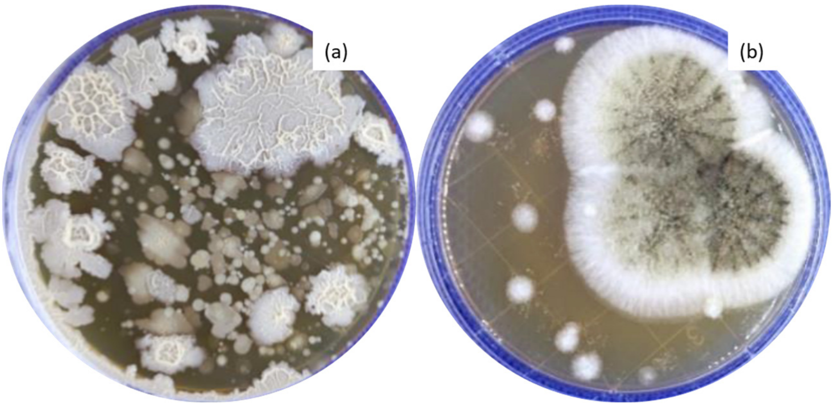

Figure S1. (a) Colonies growth on MEA and (b) MEA added with chloramphenicol.

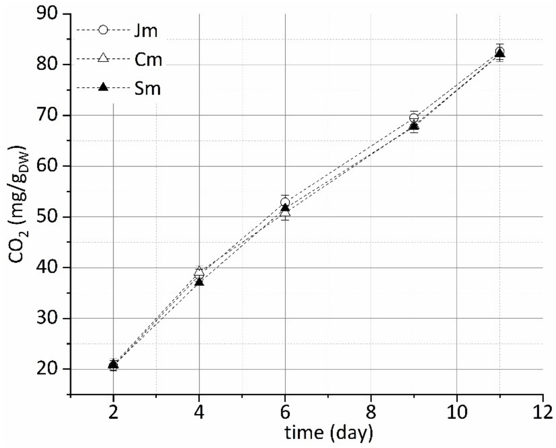

Figure S2. Cumulated CO_2 (mg/gDW) produced in microcosms with different geometry (cylindric Jm, and cone, Cm and Sm) with H hazelnuts. $p>0.05$ for all the incubation times.

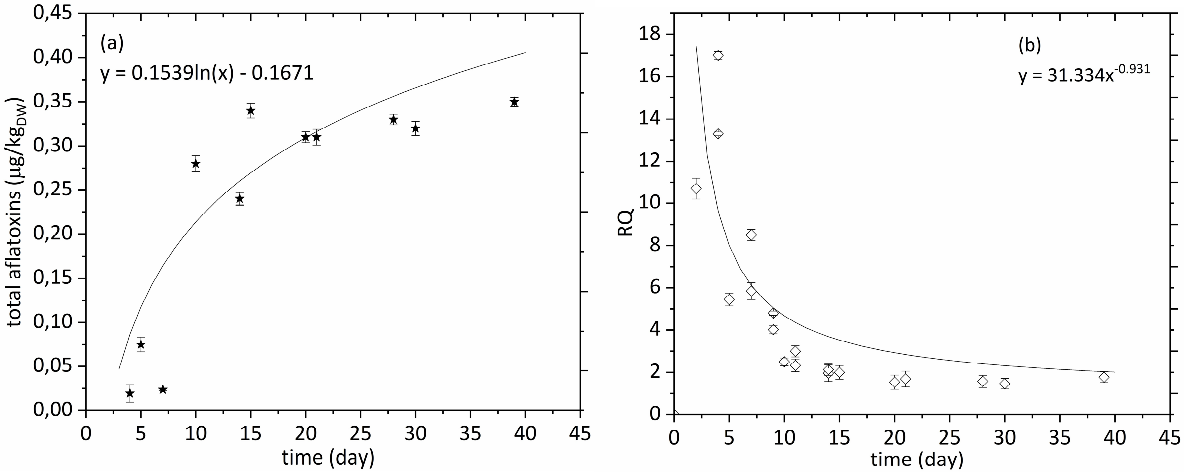

Figure S3. (a) total aflatoxin and (b) RQ values vs. time.
